# Circulating miRNAs, Small but Promising Biomarkers for Autism Spectrum Disorder

**DOI:** 10.3389/fnmol.2019.00253

**Published:** 2019-10-11

**Authors:** Salam Salloum-Asfar, Noothan J. Satheesh, Sara A. Abdulla

**Affiliations:** Neurological Disorders Research Center, Qatar Biomedical Research Institute (QBRI), Hamad Bin Khalifa University (HBKU), Qatar Foundation (QF), Doha, Qatar

**Keywords:** neurodevelopmental disorders, circulating miRNA, biomarkers, Autism Spectrum Disorder, microrna, biofluid analysis

## Abstract

Autism spectrum disorder (ASD) refers to a heterogeneous group of complex neurodevelopmental disorders characterized by social skill and communication deficits, along with stereotyped repetitive behavior. miRNAs, small non-coding RNAs that have been recognized as critical regulators of gene expression, play a key role in the neurodevelopmental transcriptional networks of the human brain. Previous investigations have proven that circulating miRNAs open up new possibilities for the emerging roles of diagnostic and prognostic biomarkers in human disorders and diseases. Biomarker development has been progressively becoming more recognized as a cornerstone in medical diagnosis, paving the way to drug discoveries and limiting the progression of various diseases. Due to the complexity of ASD, considerable endeavors have either unsuccessfully identified biomarkers for the disorder or have not yet been established. Cell-free circulating miRNAs in biofluids are extraordinarily stable and considered to represent the next-generation of clinical, non-invasive, biomarkers for many pathologies including neurological and neurodevelopmental disorders. Here, we conducted a review of all peer-reviewed articles addressing the circulating profiles of miRNAs, mostly performed in serum and saliva samples in individuals with ASD.

## Background and Clinical Aspects

Autism Spectrum Disorder (ASD) is a multi-faceted, pervasive, neurodevelopmental disorder that tends to reveal itself at around 18–36 months of age (Brian et al., 2008). Unfortunately, no clear etiology for the disease exists. Among the plethora of symptoms that are associated with it, the more common ones include; pragmatic aspects in language development (Matson et al., 2010), an absence in symbolic play (Thiemann-Bourque et al., 2012), an impairment in their ability to socialize (Kasari and Patterson, 2012), and the presence of repetitive or ritualistic behaviors (Greaves et al., 2006). Furthermore, ASD has been shown to associate itself frequently with comorbidities such as epilepsy (Besag, 2018), intellectual disabilities (Mpaka et al., 2016), immune dysfunction (Hughes et al., 2018), hyperactivity (Lyall et al., 2017), and gastrointestinal disorders (Chaidez et al., 2014). Furthermore, studies have aimed to understand whether genetic damage, or mediated epigenetic factors contribute to the existence of these symptoms. Consequently, a lack of common consensus has driven researchers to understand the fundamentals of the disorder and to further invest in developing better diagnostic criteria.

Remarkably, there has been a general rise in the prevalence of ASD. In 2018, the Centers for Disease Control and Prevention (CDC) has identified that ASD affects approximately 1 in 59 children around the world, a notable rise from approximately 1 in 166 in 2004. Furthermore, no common consensus for the reasons behind this drastic increase exists. Some have attributed it to an increase in a consciousness of the disorder and better diagnostic criteria, while others argue that it is due to an actual escalation in prevalence or a combination of all these factors.

Biomarker development has been progressively becoming more recognized as a cornerstone in disease diagnosis, paving the way to drug discoveries and limiting the progression of various diseases (Kalia, 2013; Blaus et al., 2015). Due to the complexity of ASD, considerable endeavors have unsuccessfully identified biomarkers for the disorder (Gandal et al., 2010; Goldani et al., 2014; Loth et al., 2016). More importantly, the need for early identifiers is crucial, as it has been previously shown that early intervention, whether through medication or behavioral therapy, can eliminate some symptoms and improve their quality of life. Consequently, there is an urgent need to develop novel approaches that aim at discovering reliable biomarkers for the early diagnosis or prediction of ASD (Shenoy et al., 2017).

Non-coding RNAs (ncRNAs) have exponentially drawn much attention in humans and have been extensively studied as a potential source of diagnostic and prognostic biomarkers (Trzybulska et al., 2018). The enormous heterogeneity between ncRNAs has been previously described. According to their average sizes, two major categories have been established; the first of which is comprised of small non-coding RNAs, including miRNAs, small interfering RNAs (siRNAs), PIWI-interacting RNAs (piRNAs), Alu RNAs, and others, and the second group consisting of long non-coding RNAs (lncRNAs), defined with lengths exceeding 200 nucleotides that are not translated into protein (Kopp and Mendell, 2018).

Recently, there has been a significant investment in improving transcriptomic approaches, such as RNA-seq and other detection methods, to further understand the functional roles of ncRNAs in various diseases and disorders (Wang et al., 2012). This review explores the role of miRNAs, pointing to their potential to become reliable biomarkers and therapeutic tools for ASD. The general concepts and critical challenges in profiling and identifying miRNAs are discussed in detail in the next sections.

## Introduction to miRNAs

miRNAs are evolutionarily conserved and constitute a phylogenetically extensive group of endogenous small (∼20–22 nts) ncRNAs that mainly arbitrate the post-transcriptional modification in gene expression (Van Rooij and Kauppinen, 2014). In 1993, the discovery of the first non-coding antisense RNA sequence lin-4 by Ambros and Ruvkun groups in *Caenorhabditis elegans* revolutionized the field of molecular biology with a deeper understanding of several human pathologies and its contribution to personalized medicine (Mellios and Sur, 2012; Van Rooij and Kauppinen, 2014; O’Brien et al., 2018). Ever since its discovery, the miRBase database has been established (Griffiths-Jones, 2004). This comprehensive, searchable platform highlights published miRNA sequences and confers 38,589 entries of miRNAs from 271 organisms with 1,917 annotations in the human genome (Kozomara et al., 2018). Approximately two-thirds of human mRNAs are predicted to be regulated by miRNAs (Hicks and Middleton, 2016), thus each mRNA could potentially have hundreds of miRNA targeted sites and a single miRNA can regulate different mRNAs (Huntzinger and Izaurralde, 2011; Fregeac et al., 2016).

Despite being short RNAs not capable to encode proteins, miRNAs hold important structural, regulatory, and catalytic functions (Pillai, 2005). While previous investigations have reported significant genetic components to ASD, there is still a need to identify a single gene variant that accounts for more than 1% of its incidences (Griesi-Oliveira and Sertié, 2017). Alternatively, the post-transcriptional mechanisms regulated by miRNAs fine-tune gene expression without altering the genetic code by targeting the 3′ untranslated region (UTR) of the mRNAs and therefore degrade mRNA or inhibit its translation (Mellios and Sur, 2012; Fregeac et al., 2016; Hicks and Middleton, 2016). Conversely, studies have also confirmed miRNAs targeting other regions like 5′ UTR, gene promoters and coding sequence (O’Brien et al., 2018). Furthermore, miRNAs have been described to play crucial roles in developmental and metabolic mechanisms in addition to cellular processes such as apoptosis, proliferation, and differentiation (Fregeac et al., 2016; Hicks and Middleton, 2016). Previous studies have highlighted their effects on spatial localization or compartmentalization of protein translation in axons, dendrites and synapses (Tonelli et al., 2008; Im and Kenny, 2012). This contributes to their influential capacity toward brain development (including neurogenesis and neuronal migration), and synapse formation (Wang et al., 2012; Hicks and Middleton, 2016), two aspects of extensive investigation in the quest to understand the mechanisms behind ASD. Furthermore, dysregulation of miRNAs have been shown to alter behavior and cognition in other neuropsychiatric disorders (Wang et al., 2012), further supporting its potentially significant role in ASD.

## miRNA Biogenesis and Function

miRNA genes are either found in inter- or intra- genic regions. Almost half of the presently annotated miRNAs are intragenic (mostly processed from introns and few exons of protein coding genes), while the other miRNAs are intergenic (regulated by their own promoters and independently transcribed of a host gene) (Fregeac et al., 2016; O’Brien et al., 2018).

**FIGURE 1:**
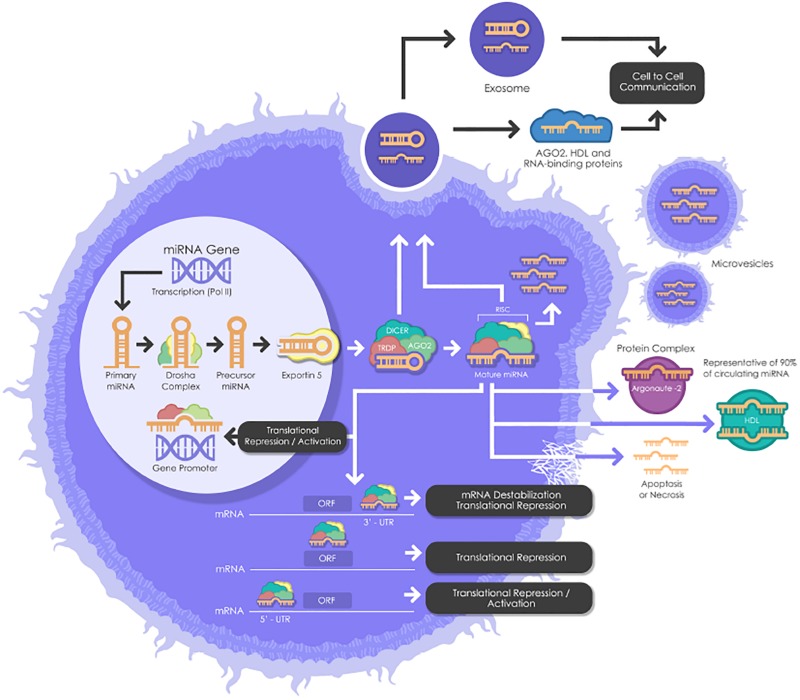
A schematic biogenesis pathway, function and mechanisms of secretion of miRNA. miRNA biogenesis begins in the nucleus where miRNA genes are transcribed as primary miRNAs (pri-miRNAs) by RNA polymerase II (Pol II). Pri-miRNAs are processed by the RNase III endonuclease, Drosha, and its cofactor, Dgcr8 into smaller stem-looped structures known as precursor miRNAs (pre-miRNA). Then these pre-miRNAs are exported to the cytoplasm by exportin 5 (XPO5) and further processed by Dicer, a ribonuclease III (RIII) enzyme that produces the mature miRNAs. Mature miRNAs are incorporated into RISC (RNA-induced silencing complex), which contains Dicer and Argonaute (AGO) proteins, to produce target mRNA degradation or mRNA translational repression or activation. miRNAs can be secreted from living cells into the extracellular environment (such as blood vessels or other biofluids) via the following mechanisms: (1) via exosomes; (2) via microvesicles; (3) as protein-miRNA complex (AGO2) (which represents 90% of circulating miRNAs) and lipoprotein complex (such as HDL); and (4) passive secretion through apoptosis or necrosis. pri-miRNAs, Primary miRNAs; Pol II, RNA Polymerase II; pre-miRNAs, miRNAs precursor; DGCR8, DiGeorge syndrome critical region gene; RISC, RNA-induced silencing complex. AGO, Argonaute; HDL, High-density lipoprotein.

miRNAs need to undergo several maturation steps (biogenesis) to become functionally active. miRNA biogenesis begins in the nucleus where miRNA genes are transcribed by RNA polymerase II to form the “primary miRNAs,” or “pri-miRNAs” containing cap structures as well as poly(A) tails. Approximately 25% of miRNAs form clusters (one long transcript) and are transcribed collectively in a one long polycistronic transcript pri-miRNA (Figure 1; Fregeac et al., 2016). In a canonical pathway (Figure 1), the microprocessor complex that consists of two subunits; a ribonuclease III enzyme, Drosha and RNA binding protein DiGeorge Syndrome Critical Region 8 (DGCR8) cleaves the newly transcribed primary miRNAs (pri-miRNAs) to form the precursor miRNAs (pre-miRNAs). Pre-miRNAs that are approximately 70 nucleotides in length are transported to the cytoplasm by EXPORTIN/RanGTP complex. However, each miRNA is processed independently when numerous miRNA precursors are present in the same transcript. Then, Dicer, known as endoribonuclease RNAse III-like, interacts with TRBP (RNA binding protein) and cleaves the hairpin loops of the pre-miRNAs, resulting in a duplex of mature miRNAs. This duplex is dissociated and the functional strand of the mature miRNAs undergoes gene silencing by RNA-Induced Silencing Complex (RISC); ARGONAUTE proteins (AGO 1-4), Dicer and TRBP binding complex. Only one strand of the mature miRNA is generally incorporated, while the other, with central mismatches or non-AGO loaded miRNAs, are degraded (Fregeac et al., 2016; O’Brien et al., 2018). The direction of the miRNA strand confers the name of the mature miRNA.

However, cells are also able to produce functional miRNAs using alternative non-canonical biogenesis pathways. Dicer is primordial for miRNAs both canonical and non-canonical biogenesis, while non-canonical miRNAs can be generated independently of Drosha and Dgcr8.

Thus far, most functional investigations on miRNAs are based on either a disruption in their biogenesis pathways, in turn effecting their expression, or by silencing selectively chosen single miRNAs within a particular region of an organ or cell type (Kleaveland et al., 2018). Both investigative strategies have yielded valuable information towards our understanding of miRNA function in physiology and pathological conditions (Wang et al., 2012).

## miRNA Roles Beyond Gene Expression Control: Circulating miRNAs (ci-miRNAs)

**FIGURE 2:**
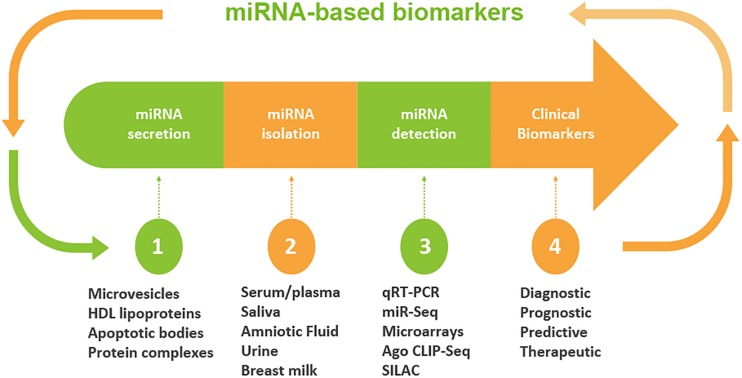
Workflow of circulating miRNA discovery in biofluids as biomarkers. A schematic diagram that shows miRNA secretion into different types of biofluids, miRNA extraction and detection approaches. Clinical decisions may be made based on the expression levels of miRNAs. The profile of miRNA is used to determine the diagnosis and/or prognosis of the patient. On the basis of diagnostic and prognostic utilities, a clinical decision can be made for the targeted and personalized medicine.

Although miRNAs were originally identified largely in cellular microenvironments, evidence for the cell-free circulating miRNAs were recognized when Chim et al. in 2018 distinguished miRNAs of placental origin from that of plasma in pregnant women (Chim et al., 2008). The extracellular microenvironments that house miRNAs include those derived from blood origins such as plasma and serum, and other biofluids including tears, saliva, urine, colostrum, breast milk, peritoneal fluid, amniotic fluid, cerebrospinal fluid, seminal fluid, synovial fluid, pleural fluid, bronchial lavage, and follicular fluid (Figure 2; O’Brien et al., 2018).

miRNAs are not only limited to be endogenous regulatory elements; a plethora of recent studies have demonstrated that miRNAs may serve as emerging biomarkers in body fluids and thus suggests the expanded role of influencing mechanisms outside the tissues in which they were originally transcribed. Contrary to intracellular miRNAs, extracellular/circulating miRNAs, detected in diverse fluids, are highly stable and do not degrade if kept at room temperature for about 4 days. Additionally, their resilience allows them to withstand adverse conditions like high/low pH, multiple-freeze thaw cycles and boiling temperatures (O’Brien et al., 2018). Previous studies have recognized that their notable stability is due to either miRNA encapsulation within microparticles like exosomes, microvesicles, and apoptotic bodies or as a result of solely RNA-binding proteins such as lipoprotein complexes like high-density lipoproteins (HDL), Argonaute2 (Ago2), and nucleophosmin I (Tkach and Thery, 2016). This, in turn, expands their reach, altering gene expression in distant tissues (Creemers et al., 2012; Min and Chan, 2015).

## Cell Free ci-miRNAs as Biomarkers

Cell-free ci-miRNAs mediate as signaling molecules for cellular communication and have been extensively accounted as potential biomarkers for several diseases (Shah et al., 2017; Paul et al., 2018). The miRNA expression profiles of certain diseased tissue and fluids are distinct from those of adjacent normal ones. As a consequence, disease-specific miRNA signatures can serve as diagnostic biomarkers. Furthermore, the discovery of miRNAs in biofluids unwraps the chance of using them as biomarkers in the screening of numerous diseases including cardiovascular diseases (Navickas et al., 2016), diabetes (Jiménez-Lucena et al., 2018), cancer (Yuan et al., 2018) as well as neurodegenerative disorders (Nussbacher et al., 2015; Sheinerman et al., 2017).

## miRNA Expression Profile in the Central Nervous System and Neurological Disorders

miRNAs are highly expressed in the central nervous system being around 70% of all miRNAs (Cao et al., 2016). During development, the brain changes radically. and miRNA expression also fluctuates in the brain during childhood. Remarkably several miRNAs have been described as vital triggers for normal brain development and aberrant expressions of these miRNAs are related to nerve-related diseases (Chen and Qin, 2015). Many miRNAs display dynamic and specific temporal and spatial patterns of expression during brain development and in adult brain. Some of the examples include; let-7 miRNA family that are found highly expressed in the developing mammalian brain and miR-9 that can attenuate amyloid-β inducing synaptotoxicity and may play a role in Alzheimer’s disease (Ziats and Rennert, 2014).

Under such premises, here, we conducted a systematic literature review of the most prominent circulating miRNAs as biomarkers in ASD.

## Ci-miRNAs as Predictive Biomarkers in ASD

**FIGURE 3:**
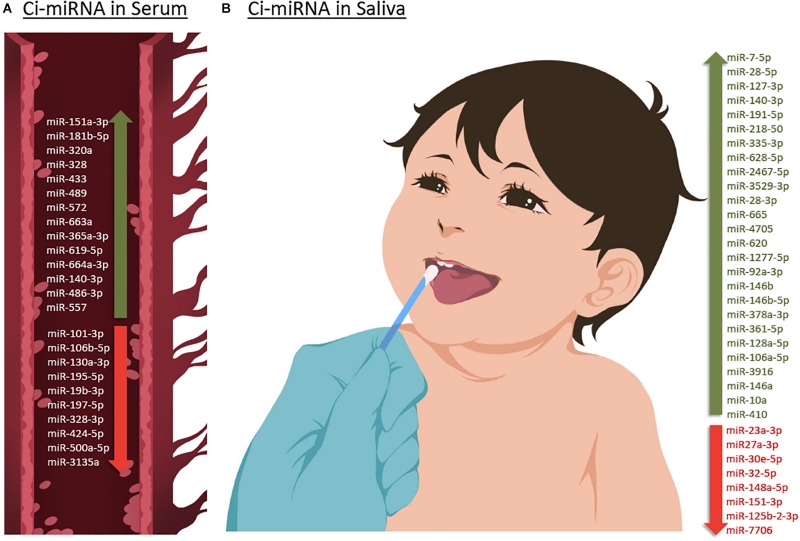
Up- or down-regulated circulating miRNA (Ci-miRNA) described in serum **(A)** and saliva **(B)** samples in ASD individuals. **(A)** Representative blood vessel with circulating miRNA reported in the literature. **(B)** Representative methodology of saliva collection from children Nucleic Acid Stabilizing Swab with all salivary miRNA reported in the literature. Green arrow pointing upwards refers to miRNA that are described to be upregulated in serum and saliva in ASD individuals. While red arrow pointing downward refers to miRNA that are found to be downregulated.

Although the research on ASD is progressively and exponentially growing, only 8 papers have explored the use of circulating miRNAs (ci-miRNAs) in biofluids (Table 1) such as in serum (Vasu et al., 2014; Cirnigliaro et al., 2017; Kichukova et al., 2017; Popov et al., 2018; Yu et al., 2018) and in saliva (Hicks et al., 2016, 2018, 2019; Figure 3). Moreover, from the few investigations, only the three studies performed using saliva specimens incorporated a high-throughput approach (RNA-seq).

**TABLE 1:** Circulating miRNAs as biomarkers for ASD.

**Sample type**	**Number**	**M:F ratio**	**Age (years)**	**RNA extraction**	**miRNA profiling and validation**	** miRNAs**		**References**
						** Upregulated**	**Downregulated**	
Serum	55 ASD 55 NC	48:7 48:7	6–16 6–16	Qiagen miRNeasy Serum/Plasma kit	miRNA PCR array and qRT-PCR	miR-151a-3p, miR-181b-5p, miR-320a, miR-328, miR-433, miR-489, miR-572, miR-663a	miR-101-3p, miR106b-5p, miR-130a-3p, miR-195-5p, miR-19b-3p	Vasu et al., 2014
	30 ASD 30 NC	24:6 24:6	3—1 3–11	PAXgene blood miRNA kit	qRT-PCR	miR-365a-3p, miR-619-5p, miR-664a-3p	miR-197-5p, miR-328-3p, miR-424-5p, miR-500a-5p, miR-3135a	Kichukova et al., 2017
	30 ASD, 24 TS 25 ASD + TS 25 NC	22:8 21:3 25:0 16:9	3–13 3–13 3–13 3–13	Qiagen miRNeasy Serum/Plasma kit	TLDA and qRT-PCR	miR-140-3p	N/A	Cirnigliaro et al., 2017
	20 ASD 23 NC	18:2 20:4	3–9 3–9	mirVana PARIS Kit	microarray and qRT-PCR	miR-486-3p, miR-557	N/A	Yu et al., 2018
	30 ASD 30 ASD	24:6	3–11	PAXgene blood miRNA kit	qRT-PCR	N/A	miR-3135a, miR-328-3p	Popov et al., 2018
Saliva	24 ASD 21 NC	19:5 16:5	5–13 4–14	Standard Trizol method, followed by a second round of purification using the RNeasy mini column (Qiagen)	RNA-seq	miR-7-5p, miR-28-5p, miR-127-3p, miR-140-3p, miR-191-5p, miR-218-50, miR-335-3p, miR-628-5p, miR2467-5p, miR-3529-3p	miR-23a-3p, miR27a-3p, miR-30e-5p, miR32-5p	Hicks et al., 2016
	238 ASD 218 NC	183:55 168:50	1–4 1–4	Isolation of epithelial or exosomal RNA	RNA-seq	miR-92a-3p, miR-146b, miR-146b-5p, miR-378a-3p, miR-361-5p, miR-128a-5p, miR-106a-5p, miR-3916, miR-146a, miR-10a, miR-410	N/A	Hicks et al., 2018
	187 ASD 125 TD 69 DD 62 NC	161:26 75:49 48:21	2–6 2–6 2–6	Standard Trizol method, followed by a second round of purification using the RNeasy mini column (Qiagen)	RNA-seq	miR-28-3p, miR-665, miR-4705, miR-620, miR-1277-5p	miR-148a-5p, miR-151-3p, miR-125b-2-3p, miR-7706	Hicks et al., 2019

*M:F ratio, Male:Female ratio, ASD, Autism Spectrum Disorder; vs., versus; TS, Tourette Syndrome; NC, Unaffected Control, TD, ASD from peers with typical development, DD, non-autism developmental delay, TLDA, TaqMan Low Density Array.*

## Ci-miRNAs in Serum as Asd Biomarkers

Six years after the discovery that miRNAs are circulating in the blood (Mitchell et al., 2008) and have the potential to become biomarkers in cancer, Vasu et al. (2014) study was the first to describe their role as potential biomarkers in ASD (Table 1). The authors screened serum samples from 55 children with ASD and 55 age- and gender-matched controls. Compared to controls, 13 miRNAs were specifically differentially dysregulated in ASD subjects. They found that miR-151a-3p, miR-181b-5p, miR-320a, miR-328, miR-433, miR-489, miR-572, and miR-663a were downregulated, while miR-19b-3p, miR-27a-3p, miR-101-3p, miR-106-5p, miR-130a-3p, and miR-195b-5p were upregulated. Furthermore, only five miRNAs, miR-181b-5p, miR-320a, miR-572, miR-19b-3p and miR-130a-3p, shown good predictive value for discriminating ASD subjects. Additionally, a subsequent miRNA target enrichment analysis identified 18 essential neurological pathways that contributed to axon guidance, TGF-beta signaling, MAPK signaling, regulation of actin cytoskeleton, oxidative phosphorylation, and mTOR signaling (Hoeffer and Klann, 2010) among others. It is important to bare in mind, that since ASD is an early-onset disorder, the sample used in these studies were from individuals ranging 6–16 years of age (Table 1), and hence calls for additional studies on subjects within a lower age range.

A few years later, a second study on ci-miRNAs serum miRNAs was performed by Kichukova et al. (2017) and found eight differentially dysregulated miRNAs in ASD subjects when compared to control NC (Table 1; Kichukova et al., 2017). miR-365a-3p, miR-619-5p, and miR-664a-3p were upregulated in ASD individuals, whereas miR-197-5p, miR-328-3p, miR-424-5p, miR-500a-5p, and miR-3135a were downregulated. Some specific downstream targets of the differentiated miRNAs, miR-619-5p and miR-328-3p, were included in the study, such as those genes related to mediation of the influx of calcium ions into the cell upon membrane polarization and calcium channel. In comparison to the previous study performed by Vasu et al. (2014), the range age of ASD individuals was lower, between 3 and 11 years (Table 1).

None of the previous studies focused on other comorbidities with autism. The third study conducted by Cirnigliaro et al. (2017), aimed to analyze the comorbid disorder (Rapanelli et al., 2017) Tourette Syndrome (TS) separately and in combination with ASD, using high-throughput approach, TLDA (Taqman Low Density Array) technology that profiled the serum expression of 754 miRNAs. They found that miR-140-3p was significantly upregulated in ASD individuals compared to controls. However, no expression differences of miR-140-3p was noted when comparing TS to controls, TS + ASD to controls, and TS + ASD to TS. Their data suggested that miR-140-3p could be used as a new, potential and a serum biomarker in the discrimination of healthy individuals from those that have ASD. This, in turn, further supports the use of different behavioral based diagnostic tools for the two neurodevelopmental disorders, ASD and TS + ASD (Cirnigliaro et al., 2017).

The study of Popov et al. (2018), showed differential expression changes of miR-3135a and miR-328-3p in the serum of ASD individuals using qRT-PCR technology. Remarkably these miRNAs were previously described to play a role in the etiopathogenesis of several neurological disorders (Provost, 2010; De Felice et al., 2012). Their study showed that miR-3135a and miR-328-3p could discriminate between ASD individuals and controls as previously demonstrated by Kichukova et al. (2017) being downregulated in their cohort. Using KEGG (Kyoto Encyclopedia of Genes and Genomes) pathway analysis, some of the target genes of miR-3135a and miR-328-3p are involved in neurodegenerative diseases, but their involvement in ASD is yet to be clarified (Popov et al., 2018).

Another study by Yu et al. (2018) showed that miR-486-3p and miR-557 are upregulated in serum samples of ASD compared to controls. Increasing evidence suggests that mutations in ARID1B, AT-rich interactive domain-containing protein 1B, are associated with ASD, which has an important role in neuronal differentiation and central nervous system maturation in brains (Yu et al., 2018). Their study revealed that miR-486-3p may play a significant role in ASD development by targeting ARID1B. The authors have shown that miR-140-3p, previously described to be upregulated in ASD individuals by Cirnigliaro et al. (2017) in serum and by Hicks et al. (2016) in saliva, was also upregulated in this cohort based on their microarray profiling results although not validated by qRT-PCR.

## Ci-miRNAs in Saliva as ASD Biomarkers

As aforementioned, similar to ci-miRNAs in serum, ci-miRNAs can be detected in saliva samples. Furthermore, the extraction of RNA from saliva is a non-invasive method of detection that avoids uncomfortable blood draws and the anxiety that is usually associated with it. Human saliva as a diagnostic specimen avoids disturbances in the viability of the samples such as the hemolysis of blood samples. Therefore ci-miRNAs from children’s saliva specimens have emerged as promising diagnostic, prognostic and predictive biomarkers in ASD.

A preliminary study published in 2016 by Hicks et al. (2016) which involved saliva samples from 24 children with ASD, has shown 14 miRNAs to be differentially dysregulated compared to controls. More specifically, miR-7-5p, miR-28-5p, miR-127-3p, miR-140-3p, miR-191-5p, miR-218-5p, miR-335-3p, miR-628-5p, miR-2467-5p, and miR-3529-3p were upregulated in ASD subjects, whereas miR-23a-3p, miR-27a-3p, miR-30e-5p, and miR-32-5p were downregulated (Table 1; Hicks et al., 2016). Additionally, 13 out of 14 miRNAs were reported to be altered in the development of the human brain of individuals between 4 months and 23 years with ASD (Ziats and Rennert, 2014). This study also showed that expression patterns of the salivary miRNAs; miR-27a, miR-23a, and miR-628-5p were significantly correlated with neurodevelopmental measures of adaptive behavior.

Two years later (Hicks et al., 2018), the same authors described a novel non-invasive approach based on salivary poly-omic RNA measurement, which not only detects miRNAs but also piwi-interacting RNA (piRNA) and other non-coding RNA, including small nucleolar RNA (snoRNA) and long intergenic non-coding RNA (lincRNA). In this study that included 426 children, 11 miRNAs were found in abundance; miR-92a-3p, miR-146b, miR-146b-5p, miR-378a-3p, miR-361-5p, miR-128a-5p, miR-106a-5p, miR-3916, miR-146a, miR-10a, and miR-410.

However, all these miRNAs had 1,862 gene targets, and 198 (11%) of these were among the 909 ASD candidate genes. miR-106a-5p targeted the largest number of ASD candidate genes (51, 12% of total targets). Six miRNAs (6/11, 55%) were previously reported in human ASD studies (Hicks and Middleton, 2016).

Recently, Hicks et al. (2019), extended their first salivary miRNA study performed in 2016 (Hicks et al., 2016) to a multi-center, prospective, case-control study (Table 1) that enrolled a big cohort of 443 children. They aimed to explore the efficacy of salivary miRNAs by differentiating children with ASD from peers with typical development (TD) and non-autism developmental delay (DD). The study provided large-scale evidence of using salivary miRNAs to distinguish children with ASD with TD or DD. Interestingly, the authors lead a clinical trial called “A Salivary miRNA Diagnostic Test for Autism; https://clinicaltrials.gov/; NCT02832557” (Hicks et al., 2019). This emphasizes miRNA capabilities which extend far beyond ASD diagnosis to its use as a predictor of ASD phenotype and severity (Vasu et al., 2014).

## Limitations of Using Circulating miRNAs as ASD Biomarkers

miRNAs profiling of biofluids in ASD individuals is still certainly in its nascent stages. It is necessary to highlight two main categories of limitations; those that result from the methodology as well as those that result from lack of functional miRNA consequences.

Regarding the methodology used by different groups, the results achieved showed inconsistencies in their findings and the detection of miRNAs in serum raised several critical technical issues. More importantly, the techniques applied need to be reviewed. Variables such as the lack of protocolized extraction methods for blood and sample hemolysis need to be considered; especially those samples with a large amount of miRNAs discharged from red blood cells after hemolysis. In addition, inconsistent findings may be due to different starting samples (serum and saliva) as well as to different RNA extraction methodologies, miRNA profiling and validation (miRNA-seq, microarray, and RT-qPCR), and data normalization methods. As shown in Table 1, most of the studies are based on qRT-PCR. Indeed, the choice of a miRNA detection platform is relevant and the qRT-PCR methodology has a limited capability to detect novel miRNAs. In addition, another limitation is the discrepancy in the consistency of utilizing equal amounts of starting material, synthetic spike-in control and the lack of using approaches to detect housekeeping circulating miRNAs to normalize expression levels.

There are also potential environmental factors that alter salivary miRNA levels such as dietary restrictions and differences in dental hygiene.

In terms of functionality, the major limitation of all these studies is the lack of experimental validation studies. All reports rely on computational and *in silico* tools to initially identify promising target candidates and functionality is yet to be clarified. Indeed, these novel putative targets need further *in vitro* and *in vivo* validation at a later stage. It is notoriously difficult to study the human brain but neurons derived from Induced pluripotent stem cells and *in vitro* brain organoids might be promising models to validate of miRNA/mRNA target interactions.

## Conclusion

Autism spectrum disorder is a complex neurodevelopmental disorder with different levels of symptom severities. Given the challenges, current clinical assessments of ASD are based on subjective evaluations of clinicians. Diagnostic strategies should move away from subjectivity. This can potentially be achieved through the use of identifiable, circulating miRNA biomarkers, paving the way for more reliable diagnosis and evaluation of pathologies and disorders. Since the discovery of circulating miRNAs in 2008, a next-generation of clinical biomarker discovery has appeared as an attractive option of diagnosis and evaluation of pathologies and disorders. Each miRNA has hundreds of potential mRNA targets and thus may control entire gene networks. Furthermore, miRNAs have been stably detected in biofluids and can circulate within microvesicles, exosomes, apoptotic bodies or bound to RNA-binding proteins. In this review, we have gathered and compiled a collection of circulating cell-free miRNAs in ASD, that have the potential to be promising biomarkers for the disorder. Furthermore, the reports and studies found in the literature are limited to only serum and saliva samples. Moreover, it is important to note that circulating miRNA profiles in both serum and saliva are dysregulated in subjects with ASD, however, no consistency is present among the studies. This inconsistency may be a result of the limited sample capacity observed between studies that analyzed saliva (large cohorts) in comparison to those that focused on serum (small cohort). Moreover, it is important to appreciate that reliable miRNA results, that entail statistically significant differences between individuals with ASD and control groups, require a large sample size.

Furthermore, these studies have used different profiling and quantitative methods. In contrast to microarray, custom arrays, and qRT-PCR, only high throughput sequencing allows the discovery of new small ncRNAs as well as the quantification of all miRNAs. Currently only three out of eight studies have used this methodology of RNA-seq in saliva samples from ASD subjects (Table 1). This is a promising area of research, where state-of-the-art technologies.

Particularly, RNA-seq has become revolutionary and a powerful state-of-the-art technique that will greatly contribute to uncovering new biomarker discovery. However, the biological significance behind the huge amount of data generated should be analyzed, interpreted, and verified using not only computational tools and algorithms but also *in vitro* or *in vivo* models to interpret each miRNA interaction with ASD target genes and its incidence.

Pathway interactions among putative miRNA targets are based on algorithms and computational tools, making these reports mainly descriptive. Further functional studies and *in vivo* models such as human brain organoids are useful to critically evaluate the molecular mechanisms behind the interaction between miRNAs and their mRNA targets reported and its contribution to ASD.

The actual diagnosis of ASD and comorbid disorders relies on behavioral assessment. However, miRNAs can contribute to define biologically homogeneous subgroups in ASD. Cirnigliaro et al. (2017) tried this approach and attempted to discriminate the comorbid disorder Tourette syndrome separately and in combination with ASD using miRNAs.

Further studies are needed to identify how miRNAs can: (1) predict ASD risk prior to the onset of behavioral abnormalities improving early diagnosis of ASD, (2) expect the developmental trajectory of children, and (3) predict response to treatment and risk for severe adverse reactions to therapy.

## Ethics Statement

This manuscript involves a review and analysis of current literature collected in preparation for the study “Identifying Potential Biomarkers for Autism Spectrum Disorder,” which has been approved by the Institutional Review Board at Qatar Biomedical Research Institute, Doha, Qatar. According to the publications on which this review was based, informed consent or assent was obtained from all participants or their parents. In the case of minor children unable to provide informed assent, parental consent was obtained.

## Author Contributions

All authors performed the research review. SS-A and SAA wrote and reviewed the manuscript.

## Conflict of Interest

The authors declare that the research was conducted in the absence of any commercial or financial relationships that could be construed as a potential conflict of interest.
